# RIC-3 differentially modulates α4β2 and α7 nicotinic receptor assembly, expression, and nicotine-induced receptor upregulation

**DOI:** 10.1186/1471-2202-14-47

**Published:** 2013-04-15

**Authors:** Alejandro Dau, Pragya Komal, Mimi Truong, Geoff Morris, Gareth Evans, Raad Nashmi

**Affiliations:** 1 Department of Biology, University of Victoria, Station CSC, PO Box 3020, Victoria, BC, V8W 3N5, Canada; 2 Department of Biochemistry and Microbiology, University of Victoria, Victoria, BC, Canada

**Keywords:** Nicotinic acetylcholine receptors, Ligand-gated ion channels, RIC-3, FRET, Nicotine

## Abstract

**Background:**

Recent work has shown that the chaperone **r**esistant to **i**nhibitors of acetyl**c**holinesterase (RIC-3) is critical for the folding, maturation and functional expression of a variety of neuronal nicotinic acetylcholine receptors. α7 nicotinic receptors can only assemble and functionally express in select lines of cells, provided that RIC-3 is present. In contrast, α4β2 nicotinic receptors can functionally express in many cell lines even without the presence of RIC-3. Depending on the cell line, RIC-3 has differential effects on α4β2 receptor function – enhancement in mammalian cells but inhibition in *Xenopus* oocytes. Other differences between the two receptor types include nicotine-induced upregulation. When expressed in cell lines, α4β2 receptors readily and robustly upregulate with chronic nicotine exposure. However, α7 nicotinic receptors appear more resistant and require higher concentrations of nicotine to induce upregulation. Could the coexpression of RIC-3 modulate the extent of nicotine-induced upregulation not only for α7 receptors but also α4β2 receptors? We compared and contrasted the effects of RIC-3 on assembly, trafficking, protein expression and nicotine-induced upregulation on both α7 and α4β2 receptors using fluorescent protein tagged nicotinic receptors and Förster resonance energy transfer (FRET) microscopy imaging.

**Results:**

RIC-3 increases assembly and cell surface trafficking of α7 receptors but does not alter α7 protein expression in transfected HEK293T cells. In contrast, RIC-3 does not affect assembly of α4β2 receptors but increases α4 and β2 subunit protein expression. Acute nicotine (30 min exposure) was sufficient to upregulate FRET between α4 and β2 subunits. Surprisingly, when RIC-3 was coexpressed with α4β2 receptors nicotine-induced upregulation was prevented. α7 receptors did not upregulate with acute nicotine in the presence or absence of RIC-3.

**Conclusions:**

These results provide interesting novel data that RIC-3 differentially regulates assembly and expression of different nicotinic receptor subunits. These results also show that nicotine-mediated upregulation of α4β2 receptors can be dynamically regulated by the presence of the chaperone, RIC-3. This could explain a novel mechanism why high affinity α4β2 receptors are upregulated in specific neuronal subtypes in the brain and not others.

## Background

Nicotinic acetylcholine receptors (nAChRs) are pentameric ligand-gated cation channels that are activated by the endogenous agonist acetylcholine and the pharmacological agent of tobacco, nicotine. There are 17 different nicotinic subunits found in vertebrates, 12 of which are the neuronal variety (α2-α10 and β2-β4). The most prevalent neuronal nicotinic receptor is the heteromeric α4β2* receptor subtype (* denotes that the receptor may comprise distinct additional subunits) and is the major receptor subtype in the brain with high affinity to nicotine. This receptor upregulates with chronic nicotine exposure in a variety of *in vitro* and *in vivo* models including cultured cell lines[1-4], rodents’ brains [5-7] and the brains of smokers [8]. However, the mechanism of nicotinic receptor upregulation is not clearly delineated although many mechanisms have been proposed [1-4,9,10]. The homomeric α7 receptor is the next most prevalent nAChR in the CNS with low sensitivity to nicotine. These receptors are more resistant to nicotine-induced upregulation, though α7 receptor upregulation can be attained with higher concentrations of nicotine in cell culture [11-13] and in more limited brain regions of receptor upregulation in rodents with *in vivo* nicotine administration [14]. Furthermore, until recently unlike α4β2 receptors, which can be functionally expressed in many cell lines, α7 can only be functionally expressed in select cell lines [15-17].

A breakthrough in the field occurred when the *ric-3* gene was discovered in mutant *Caenorhabditis elegans*, which were resistant to inhibitors of acetylcholinesterase [18,19]. The gene product, RIC-3, which is a transmembrane protein with cytoplasmic coiled-coil domains [20], enhances functional expression of nicotinic receptors, 5-HT3 receptors but not GABA nor glutamate ligand-gated ion channels [17,19,21-23]. RIC-3 is an obligatory chaperone protein for maturation, assembly and surface trafficking of α7 receptors [20-22]. It was also reported that RIC-3 can interact with the heteromeric α4β2 receptors [17,19]. However, the role of RIC-3 in influencing α4β2 receptor functional expression appears more complex due to conflicting reports. While some studies showed that RIC-3 can inhibit function of α4β2 receptors when coexpressed in *Xenopus* oocytes [24], another showed that in mammalian cells RIC-3 can enhance the function of α4β2 nAChRs [17].

In the present study, we compared and contrasted the effects of RIC-3 in modulating assembly, trafficking and protein expression between α4β2 and α7 nAChRs in HEK293T mammalian cells, using nicotinic receptors tagged with fluorescent proteins. We also examined whether the presence of RIC-3 may alter nicotine-induced receptor upregulation for α4β2 nAChRs and whether the coexpression of RIC-3 can facilitate nicotine-induced upregulation for α7 receptors, which are relatively resistant to upregulation as compared to α4β2. We found that RIC-3 increased α7 receptor assembly and trafficking to the surface but did not alter α7 protein expression. In contrast, RIC-3 did not affect α4β2 nAChR assembly but increased α4 and β2 protein expression. Interestingly, acute application of nicotine for 30 min was sufficient to upregulate FRET between α4 and β2 subunits, while RIC-3 prevented nicotine-mediated increased FRET between α4 and β2. Nicotine exposure for 30 min was incapable of upregulating assembly of α7 receptors, whether in the presence or absence of RIC-3.

## Methods

### cDNA constructs

Mouse α7, α4 and β2 nAChR cDNAs were kindly provided by Jerry Stitzel (University of Michigan, Ann Arbor, MI). Human RIC-3 cDNA was provided by Neil Millar (The University College London, UK [17]). William Green kindly provided CFP-RIC-3, in which cyan fluorescent protein (CFP) is fused to the N terminus of RIC-3 (University of Chicago [22]). Venus fluorescent protein cDNA was provided by Atsushi Miyawaki (Riken Brain Science Institute, Tokyo, Japan) [25] and Cerulean fluorescent protein cDNA was provided by David Piston (Vanderbilt University Medical Center, Nashville, Tennessee)[26]. The construction of α4CFP, α4YFP (yellow fluorescent protein), β2CFP and β2YFP nAChR subunits, with the fluorescent protein inserted into the M3-M4 cytoplasmic loop, has been reported previously and they function normally in every respect [1,27,28].

Fluorescently tagged α7 nAChR subunits were produced by inserting Cerulean or Venus cDNA into the M3-M4 cytoplasmic loop of α7. A BstEII restriction site was introduced into the M3-M4 intracellular loop of α7 using site directed mutagenesis (QuikChange XL Site-Directed Mutagenesis Kit, cat# 200521, Stratagene) using the forward primer 5^′^-CT CTA CAT TGG CTT CCG AGG CGG TCA CCT CCT GGA GGG CAT GCA CTG TG -3^′^ and the reverse primer 5^′^-CA CAG TGC ATG CCC TCC AGG AGG TGA CCG CCT CGG AAG CCA ATG TAG AG-3^′^. Cerulean was PCR amplified (Expand High Fidelity Plus PCR System, cat# 03 300 242 001, Roche) with the forward primer 5^′^ - T TTT CGG TCA CCTT GAG CAG AAG CTG ATC TCA GAG GAG GAT CTG GTG AGC AAG GGC GAG GAG CTG TTC - 3^′^ and the reverse primer 5^′^ - A AAA CAG CTT CTG CTC CAT ATC ACC TGA TCG CTG CGG TGA CC CTT GTA CAG CTC GTC CAT GCC GAG - 3^′^. This introduced flanking BstEII restriction sites and an upstream c-myc epitope tag. Similarly, Venus was PCR amplified with the forward primer 5^′^ - T TTT CGG TCA CCTT TAT CCT TAT GAC GTC CCA GAC TAC GCC GTG AGC AAG GGC GAG GAG CTG TTC - 3^′^ and the reverse primer 5^′^ - A AAA GTG CAC ACG GTA AGG ATG GTA GTC TCA CGG TGA CC CTT GTA CAG CTC GTC CAT GCC GAG - 3^′^ introducing flanking BstEII restriction sites and an upstream hemagglutinin epitope tag. We performed whole-cell patch-clamp recordings on HEK293T cells transfected with α7-Venus and wildtype α7 cDNA. α7-Venus receptors function normally. They have similar maximal peak current amplitudes and dose–response relations to ACh as compared to wildtype α7 receptors (data not shown).

### Culture and transfection of HEK293T cells

Human embryonic kidney HEK293T cells (ATCC) were maintained in Dulbecco’s Modified Eagle medium (DMEM), supplemented with 10% fetal calf serum, 2 mM L-glutamine, 100 U/ml penicillin and 100 μg/ml streptomycin. Glass coverslip bottom dishes (35 mm, cat# P35G-0-14-C, MatTek Corporation) were coated for 2 hrs with 1% gelatin in PBS. Cells were plated onto the dishes and maintained in a 5% CO_2_ incubator at 37°C. Cells were grown to 40–50% confluency and then transiently transfected with Fugene Transfection Reagent (cat # PRE2311, Promega). For each dish, equimolar masses (0.4 - 0.6 μg) of α7-Venus and α7-Cerulean or α4CFP and β2YFP cDNA were mixed in 200 μl of incomplete DMEM medium (containing 2 mM L-glutamate but lacking serum and antibiotics) with 3 μl of Fugene transfection reagent (cat # PRE2311, Promega) and transfection was performed according to the manufacturer’s protocol. In experiments studying the effects of RIC-3, RIC-3 was also added to the transfection mixture with incomplete DMEM medium at 0.02:1, 0.1:1, 1:1, or 5:1 molar ratios of RIC-3 to nAChR plasmid cDNA.

### Spectral confocal imaging

Cultured HEK293T cells were imaged 2 days post-transfection in extracellular solution (ECS) maintained at 30°C using a heated stage (cat# QE-1, Warner Instruments) connected to a temperature controller (cat# TC-344B, Warner Instruments). ECS contained in mM 150 NaCl, 4 KCl, 2 MgCl_2_, 2 CaCl_2_, 10 HEPES, 10 glucose and 2 ascorbic acid (pH 7.4). For experiments that required nicotine treatment, cultured cells were incubated at 37°C for 30 min with nicotine at one of various concentrations (0.1, 1 or 10 μM) and then washed three times in warmed ECS prior to imaging. Imaging was performed on a Nikon C1si laser scanning spectral confocal inverted microscope (Eclipse Ti-E, Nikon) using a 60X oil CFI Plan Apo VC objective (1.40 N.A., 0.13mm working distance). For each cell, a lambda stack of X-Y images was collected simultaneously with one laser sweep and onto an array of 32 photomultiplier tubes over a wavelength range from 466 – 626 nm at 5 nm separation. Images were acquired at 512 × 512 pixels at 25 × 25 μm. The pixel dwell time was set at 10 μsec and the pinhole was set to large (100 μm diameter).

### Pixel based sensitized acceptor emission FRET microscopy

Pixel-by-pixel based sensitized acceptor FRET microscopy was performed according to previous reports [28-32]. It was necessary to correct for spectral bleed through (SBT) arising from the significant overlap of Venus/Cerulean or CFP/YFP excitation and emission spectra. To detect bleed through due to emission of the donor fluorophore (Cerulean or CFP) signal into the acceptor channel (Venus or YFP) (SBT_donor_), cells expressing donor fluorophore only (CFP or Cerulean) were imaged. The donor fluorophore was excited with the 457 nm line of an argon laser at 5% maximum intensity, and emission intensities were collected at both the emission peak channel of Cerulean/CFP (478 nm) and the emission peak channel of Venus/YFP (528 nm). Similarly, to detect bleed through due to partial excitation of the acceptor when exciting the donor (SBT_acceptor_), cells expressing acceptor fluorophore only (Venus or YFP) were imaged. The acceptor fluorophore (Venus or YFP) was excited at both the 457 nm line at 5% maximal intensity) and the 514 nm line (0.5% maximal intensity) of the argon laser, and emission was detected at the 528 nm channel. Bleed through ratios were calculated to be 0.15 for SBT_acceptor_ and 0.49 for SBT_donor_. The spectral gain setting was set to a value so that there were no saturated pixels.

Sensitized emission FRET efficiencies were then determined for cells expressing both donor and acceptor. The acceptor fluorphore (YFP/Venus) was excited with the 514 nm line at 0.5% maximal intensity, and acceptor emission intensity was measured at the 528 nm channel (I_acceptor_).

Then the donor fluorophore (CFP/Cerulean) was excited with the 457 nm line at 5% maximal intensity, and emission was measured at the 478 nm channel for measurement of the donor fluorescence intensity (I_donor_), and at the 528 nm channel for measurement of sensitized acceptor emission of FRET fluorescence (I_FRET_). Image analysis of FRET efficiencies was performed with ImageJ v1.43r software (http://rsbweb.nih.gov/ij/) using either the PixFRET plugin [31] to determine pixel based FRET efficiencies or using mean pixel values within a region of interest (ROI) for ROI based FRET efficiency calculations but using the same calculations as that of the PixFRET plugin. Nikon ics/ids confocal image files were opened using the ImageJ plugin, “loci_tools.jar” (http://www.loci.wisc.edu/bio-formats/imagej). Net FRET (nFRET) was calculated according to equation (1), using the bleed through ratios reported above.

(1) $$\mathrm{nFRET}=\left({\text{I}}_{\mathrm{FRET}}\right)\;–\;\left({\mathrm{SBT}}_{\mathrm{donor}}\right)\;\text{x}\;\left({\text{I}}_{\mathrm{donor}}\right)\;–\;\left({\mathrm{SBT}}_{\mathrm{acceptor}}\right)\;\text{x}\;\left({\text{I}}_{\mathrm{acceptor}}\right)$$

For all cells, the background signal was subtracted and intensity measurements were collected from regions of interest (ROI) on the cell expressing an even and unsaturated fluorescence emission signal. FRET efficiencies were calculated by the ImageJ 1.43r software, according to equation (2),

(2) $$E=1–\left({\text{I}}_{\mathrm{DA}}/{\text{I}}_{D}\right)$$

where E represents FRET efficiency, I_DA_ represents emission intensity of the donor in the presence of acceptor (I_donor_, equation (1)), and I_D_ represents emission intensity of the donor alone [33]. I_D_ was estimated by the software as the experimental donor emission intensity in the presence of the acceptor I_DA_ or I_donor_ added to the emission intensity of the sensitized acceptor emission channel (nFRET ), as shown in equation (3).

(3) $$E=1–\left({\text{I}}_{\mathrm{DA}}/\left({\text{I}}_{\mathrm{DA}}+\;\mathrm{nFRET}\right)\right)$$

**Figure 1:**
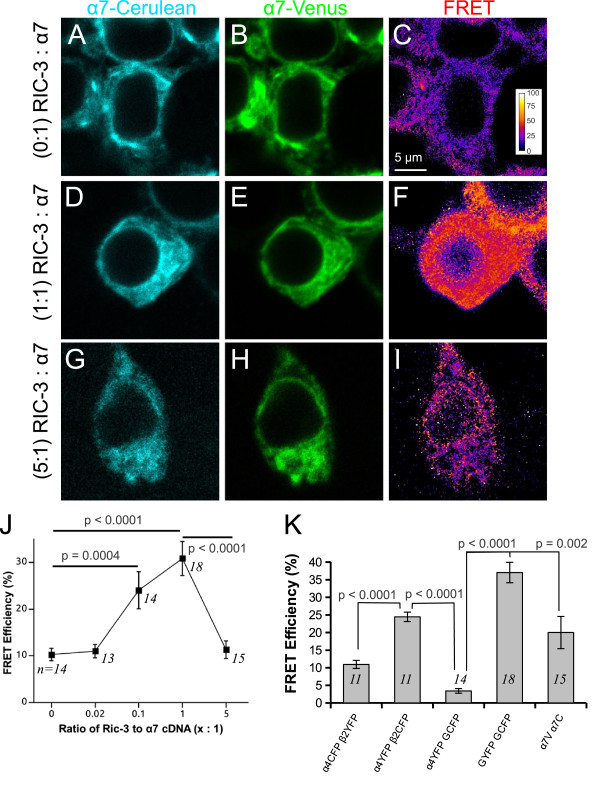
**Changes in FRET efficiency between fluorescent α7 subunits upon co-transfection with RIC-3.** Pixel based FRET was used to monitor assembly between α7-Venus and α7-Cerulean nAChR subunits. RIC-3 concentration was varied as molar ratio to total α7 plasmid: 0:1 (negative control), 0.02:1, 0.1:1, 1:1, and 5:1. Confocal images of transfected HEK293T cells showing α7-Cerulean (**A**, **D**, **G**), α7-Venus (**B**, **E**, **H**) and FRET efficiency (**C**, **F**, **I**) expression. FRET efficiency was higher in cells expressing RIC-3 at a 1:1 ratio to α7 (**F**) than cells not expressing RIC-3 (**C**) or cells expressing RIC-3 at a 5:1 ratio to α7 (**I**). (**J**) Summary plot showing that FRET efficiencies increased significantly (p < 0.0001, Kruskal-Wallis rank sum test) with increasing concentrations of RIC-3 relative to α7 but went to baseline at 5:1 ratio. There was a significant increase of FRET efficiency at 0.1:1 RIC-3 to α7 (p = 0.0004, Wilcoxon rank sum test) as compared to no RIC-3 cells and at 1:1 RIC-3 relative to α7 (p < 0.0001, Wilcoxon rank sum test). Numbers inside the plot represent the number of cells analyzed. (**K**) Control experiments validating the FRET measurements. α4YFP β2CFP shows significantly greater FRET efficiency than α4CFP β2YFP. This is expected because the transfection ratio favours an (α4)_3_(β2)_2_ stoichiometry [1] and the combination with the more acceptors (YFP) theoretically would have greater FRET efficiency. GYFP GCFP (GluClβ-YFP GluClα-CFP) are heteromeric glutamate-gated chloride channels found in invertebrates and show high FRET. Although they are members of the cys-loop family of receptors, they are not expected to assemble with any of the nicotinic receptors. Our negative control experiment, α4YFP GCFP (GluClα-CFP) shows very little FRET. A positive control experiment with RIC-3 and α7V α7C (α7-Venus α7-Cerulean) showing significant levels of FRET. Numbers inside the bars represent the number of cells analyzed.

The PixFRET plugin calculated FRET efficiency does not correct the nFRET value for the difference in relative brightness of donor and acceptor based on their respective extinction coefficients and quantum efficiencies. A set of control experiments are shown in Figure 1K validating the FRET measurements for relative comparisons of FRET changes.

### Labelling and imaging of α-bungarotoxin and epitope tag antibody binding

For surface labelling of α-bungarorotoxin sites, cells were fixed in 4% paraformaldehyde (PFA) dissolved in PBS for 10 to 30 min at room temperature. Cells were then incubated in 2 μg/ml Alexa Fluor 647 α-bungarotoxin (Fl-Bgt) (cat# B35450, Invitrogen) diluted in PBS for 30 min. For total α-bungarotoxin labelling (cytoplasmic and surface), cultured cells were fixed in 4% PFA for 30 min at room temperature followed by 5 min incubation of 0.25% Triton-X to permeabilize the membrane, and then 2 μg/ml Fl-Bgt incubation for 30 min. Between each step, cells were washed three times in PBS for 5 min intervals.

α4YFP and β2YFP subunits each contained a hemagglutinin (HA) epitope tag in addition to the fluorescent protein. For anti-HA tag antibody (rabbit polyclonal, cat# ab9110, abcam) labelling of α4YFP or β2YFP total nicotinic receptors, cells were fixed in 4% PFA in PBS for 10 min followed by 5 min incubation of 0.25% Triton-X, 30 min incubation of 10% donkey serum, 1 hr incubation of anti-HA (1:100 diluted in 3% donkey serum in PBS) and finally 1 hr incubation of CY5 conjugated donkey anti-rabbit IgG secondary antibody (1:200 diluted in 3% donkey serum in PBS). Cells were washed three times for 5 min in PBS between each step.

Measurement of α7-Cerulean/Venus or α4CFP β2YFP fluorescence was performed as previously reported for the sensitized emission experiments. Fl-Bgt was excited with a 638 nm diode laser line at 15% maximal intensity, and emission was measured at the emission peak channel (665 nm) following spectral unmixing with a 525 nm signal as a reference. CY5 was excited with a 638 nm diode laser line at 3% maximal intensity and emission measured following spectral unmixing. Images were analyzed for mean signal intensity using ImageJ v1.43r software.

### Statistical analysis

Values are reported as mean ± standard error. Significant difference (p < 0.05) between more than two groups of data meeting assumptions of normality and homogeneity of variances was analyzed by a one-way ANOVA followed by post hoc multiple pairwise analysis using a Tukey’s HSD test. If the data did not meet these assumptions a Kruskal-Wallis rank sum test was performed followed by pairwise comparisons using Wilcoxon rank sum tests. Significant difference (p < 0.05) between two groups of data was determined using a *t-*test for continuous data meeting parametric assumptions of equal variances and normality. Otherwise, a Wilcoxon rank sum test was performed for nonparametric data. We also performed the Welch two sample *t*-test for a comparison of two groups of data which were normally distributed but failed the equal variance test. For analysis of data with two independent variables, a two-way ANOVA was performed, followed by post hoc multiple pairwise analysis using a Tukey’s HSD test. All statistical analyses were performed using the R statistical computing language [34].

## Results

### RIC-3 mediates assembly between α7 subunits

FRET is a spectroscopic technique that can determine whether two fluorescently tagged molecules interact since the distance separating the donor and acceptor fluorophores must be within 100 Å for energy transfer to occur. We have previously used FRET as a spectroscopic technique to assay receptor assembly between fluorescently tagged α4 and β2 nAChR subunits in living cells [1,27]. Based on previous literature that RIC-3 is necessary for functional expression of α7 nAChRs, we performed FRET experiments of fluorescently labelled α7 nAChR subunits in the absence and various concentrations of RIC-3 to examine whether α7 subunits are assembled in the absence of RIC-3 and to what extent RIC-3 can stimulate the assembly of α7 nicotinic receptors.

To determine the influence of RIC-3 on α7 nAChR assembly, α7 subunits tagged with either Cerulean or Venus fluorescent proteins were cotransfected with RIC-3 at 0:1, 0.02:1, 0.1:1, 1:1 and 5:1 transfection ratios (RIC-3 to α7 cDNA), and sensitized FRET imaging was performed to determine the absolute FRET efficiency between subunits. Increasing expression of the RIC-3 chaperone with fluorescently tagged α7 subunits led to a significant and steady increase (p < 0.0001, Kruskal-Wallis rank sum test) in FRET efficiency (Figure 1). This increase was significant at 0.1:1 (p = 0.0004, Wilcoxon rank sum test post-hoc analysis) and at 1:1 (p = 0.0001, Wilcoxon rank sum test post-hoc analysis) transfection ratios of RIC-3 to α7 cDNA, and peaked at the 1:1 transfection ratio. The highest level of RIC-3 expression (5:1) reduced the FRET efficiency toward control value. Figure 1 shows the relative fluorescence from the donor (α7-Cerulean), the acceptor (α7-Venus) and pixel based FRET efficiency image in the control (no RIC-3), 1:1, and 5:1 molar ratios (RIC-3 to α7 cDNA). Consistent with the summary data presented in Figure 1J, the FRET signal was higher when RIC-3 was cotransfected at a 1:1 ratio than in cells that lacked RIC-3 or expressed RIC-3 at a 5:1 ratio to α7. Therefore, increasing doses of RIC-3 progressively increases assembly of α7 nAChRs peaking at a 1:1 ratio of RIC-3:α7 subunits. However, when RIC-3 outnumbers α7 then assembly of α7 nicotinic receptors decreases to baseline values similar to that found in the absence of RIC-3.

### RIC-3 increases intracellular and surface trafficking of α7 nAChRs

**Figure 2:**
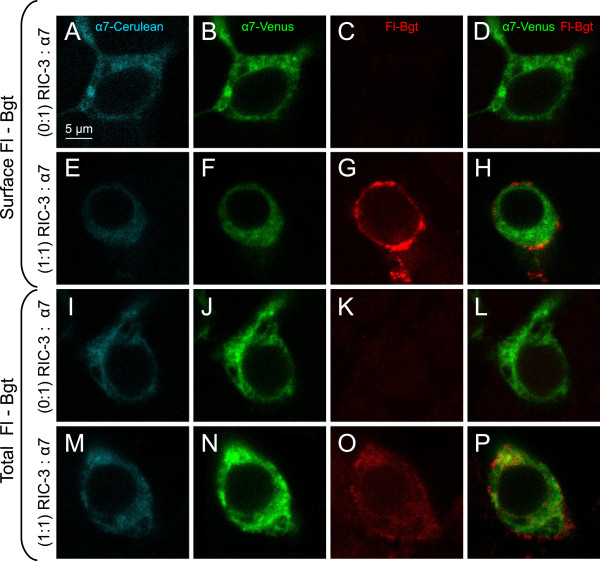
**RIC-3 increases whole cell and cell surface expression of α-Bgt binding sites.** Spectral confocal microscopy images of α-Bgt binding assays using Alexa 648 α-Bgt (Fl-Bgt) and performed under nonpermeabilizing conditions (**A**, **B**, **C**, **D**) without RIC-3, or (**E**, **F**, **G**, **H**) with RIC-3 (1:1 to α7), and permeabilizing conditions (**I**, **J**, **K**, **L**) without RIC-3 and (**M**, **N**, **O**, **P**) with RIC-3 (1:1 to α7). Emission signals from α7-Cerulean, α7-Venus, Fl-Bgt binding sites and the merged α7-Venus / Fl-Bgt images are shown for each corresponding cell. RIC-3 increases surface and intracellular α-Bgt binding sites.

**Figure 3:**
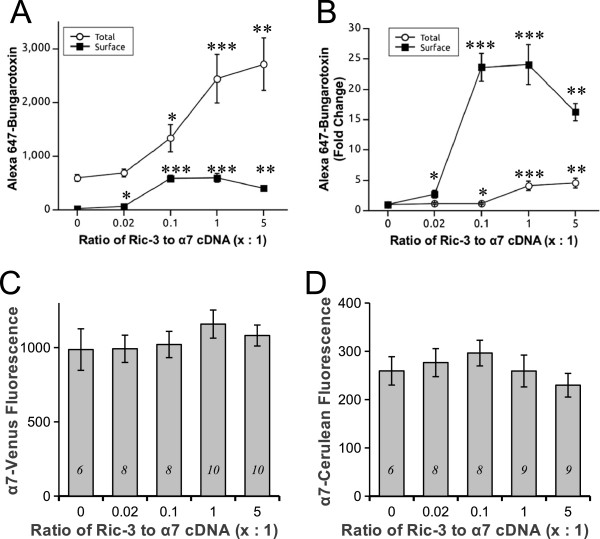
**RIC-3 increases whole cell Fl-Bgt labelling and surface trafficking of α7 receptors but does not alter protein levels.** Increasing concentrations of RIC-3 progressively augmented total Fl-Bgt binding under cell permeabilizing conditions (**A**, **B**). Fl-Bgt binding of surface α7 receptors also increased with RIC-3 but peaked at 1:1 RIC-3 to α7 and diminished at a 5:1 ratio (**A**, **B**). Total (**A**) and fold-change (**B**) of Fl-Bgt binding is shown. The surface and whole cell number of Fl-Bgt binding sites was quantified by measuring the integrated density of fluorescence intensities of Alexa 648-tagged α-bungarotoxin around the outer surface of each cell or within the cell, for non-permeabilizing and permeabilizing conditions, respectively. Significant difference levels comparing groups of RIC-3 coexpressing cells relative to no RIC-3 controls: * p < 0.05, ** p < 0.01, *** p < 0.001 Wilcoxon rank sum test post-hoc pair wise analysis. The number of cells analyzed for total receptor labelling in (**A**, **B**) are 0:1 (negative control, n = 14), 0.02:1 (n = 13), 0.1:1 (n = 14), 1:1 (n = 18), and 5:1 (n = 15). The number of cells analyzed for surface receptor labelling in (**A**, **B**) are 0:1 (n = 6), 0.02:1 (n = 8), 0.1:1 (n = 8), 1:1 (n = 11), and 5:1 (n = 11). (**C**, **D**) Mean emission intensity of the α7-Venus and α7-Cerulean fluorophores per HEK293T cell were determined at various concentrations of RIC-3. There was no significant change in either α7-Venus or α7-Cerulean fluorescent protein levels with various amounts of RIC-3 co-expressed inside the cells (p = 0.6, one-way ANOVAs; and p = 0.2, Kruskal-Wallis rank sum test, respectively).

Several studies have proposed that RIC-3 promotes surface trafficking of α7 receptors [17,22,23,35]. α-Bungarotoxin is a slowly reversible competitive antagonist that binds at the interface of adjacent α7 nAChR subunits and therefore can indicate the degree of receptor assembly. Alexa Fluor 637 α-bungarotoxin (Fl-Bgt) labelling in nonpermeabilized and permeablized conditions was performed to accurately quantify assembled α7 nAChRs both at the surface and the total cellular pool of receptors, respectively. Confocal images of control cells and cells coexpressing RIC-3 at the 1:1 ratio are shown in Figure 2. The Fl-Bgt signal was almost undetectable in the absence of chaperone, and markedly increased in intensity at a 1:1 transfection ratio. In nonpermeabilized conditions with RIC-3 coexpressed at 1:1, Fl-Bgt labelling of surface receptors displayed a ring-like labelling pattern on the perimeter of the cell. Under permeabilized conditions the total Fl-Bgt labelling appeared evenly throughout the cytoplasm of the cell, with no obvious outline of Fl-Bgt labelling near the cell surface. This indicates that although surface receptors exist, there is a large cytoplasmic pool of receptors. In general, whole cellular levels of assembled α7 nAChRs were higher than levels of surface receptors (Figure 3). The total cellular number of Fl-Bgt binding sites increased steadily with increasing levels of RIC-3 (p < 0.0001, Kruskal-Wallis rank sum test), with the highest expression at a 5:1 transfection ratio of RIC-3 to α7 cDNA (4.6 fold increase vs no RIC-3) (Figure 3). In contrast, surface Fl-Bgt fluorescence intensity steadily increased (p = 0.0003, Kruskal-Wallis rank sum test) to a peak of 24 fold at a 1:1 ratio to α7 and decreased closer to baseline at a 5:1 ratio, which paralleled our FRET data (Figure 3). These results show that RIC-3 enhances assembly and forward trafficking of α7 nAChRs to the cell surface.

### RIC-3 does not increase α7 subunit protein expression

It is possible that the increase in total α-Bgt binding sites with RIC-3 coexpression was not caused by upregulated assembly alone but also by changes in the protein levels of individual subunits. Therefore, we examined whether the increase in total Fl-Bgt binding sites was caused by an upregulated amount of α7 nAChR subunit protein. To address this, the emission signals generated by α7-Cerulean and α7-Venus were measured with various levels of RIC-3 to detect any effect of the chaperone on subunit expression levels. There were no significant changes in fluorescence intensity of both α7-Venus (p = 0.7, one-way ANOVA) and α7-Cerulean (p = 0.2, Kruskal-Wallis rank sum test), indicating that similar amounts of α7 subunits were expressed regardless of the level of expression of RIC-3. Therefore, RIC-3 stimulates α7 receptor assembly but has no effect on protein levels of individual α7 nAChR subunits.

### RIC-3 does not increase assembly between α4 and β2, but increases α4 and β2 subunit protein expression

**Figure 4:**
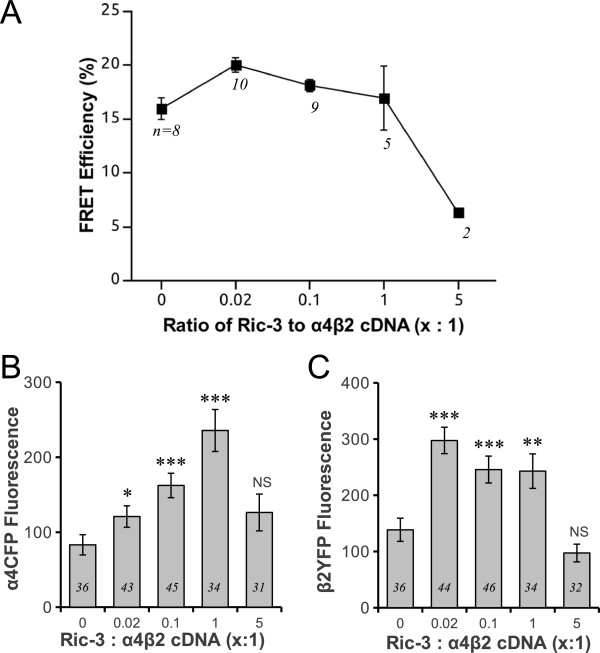
**Effect of various concentrations of RIC-3 on FRET efficiencies between α4CFP and β2YFP subunits and protein expression levels of α4CFP and β2YFP subunits.** Equimolar amounts of α4CFP and β2YFP subunits were coexpressed with various concentrations of RIC-3. The ratio of RIC-3 to α4CFP β2YFP nAChR cDNAs was varied to 0:1 , 0.02:1, 0.1:1, 1:1, and 5:1. (**A**) Quantification of FRET efficiencies between α4CFP and β2YFP subunits showed no significant change over various concentrations of RIC-3 up to 1:1 concentration (p = 0.08, one-way ANOVA). (**B**) Quantification of mean α4CFP fluorescence per cell showed a significant progressive increase with rising concentrations of RIC-3 (p < 0.0001, Kruskal-Wallis rank sum test). (**C**) The mean β2YFP fluorescence intensity per cell showed a significant increase with increasing concentrations of RIC-3 (p < 0.0001, Kruskal-Wallis rank sum test). (**B**, **C**) However, high RIC-3 concentrations at 5:1 reduced both α4CFP and β2YFP fluorescence intensities closer to baseline values. Significant difference levels comparing groups of RIC-3 coexpressing cells relative to no RIC-3 controls: * p = 0.02, ** p = 0.002, *** p < 0.0001, NS p > 0.05, Wilcoxon rank sum test post-hoc pair wise analyses.

Previous work has suggested that RIC-3 can interact with a variety of receptor subunits in addition to α7 nAChRs [17,22,23,35]. Therefore, it is important to determine whether RIC-3 specifically increases α7 nAChR assembly or whether it can also enhance assembly of α4β2 receptors, the major heteromeric nAChRs in the CNS. Using FRET measurements to quantify receptor assembly, we found that varying the amounts of RIC-3 did not significantly change the FRET efficiencies between α4CFP and β2YFP nicotinic subunits up to a 1:1 ratio of RIC-3 to α4CFP β2YFP cDNA (p = 0.08, one-way ANOVA) (Figure 4). Thus, RIC-3 has no influence on the assembly of α4β2 receptors, as is the case with α7. However, at a high RIC-3 concentration (5:1, RIC-3 : α4CFP β2YFP) there was decreased FRET between α4CFP and β2YFP, indicating fewer assembled receptors. This was similar to the effect of decreased FRET between α7-Venus and α7-Cerulean with high RIC-3 expression at 5:1.

Because RIC-3 at the moderate to low concentrations had no effect on assembly of α4β2 receptors, we expected that there would be no change in α4 and β2 subunit protein levels. To our surprise, we found that there was a significant dose–response effect of RIC-3 in increasing α4 subunit protein levels as measured by α4CFP fluorescence (p < 0.0001, Kruskal-Wallis rank sum test) (Figure 4B). There was a step-wise increase in α4CFP fluorescence from 0:1 to 1:1 ratio of RIC-3 to nAChR, with a decrease closer to baseline levels with RIC-3 expression at 5:1. Similarly, there was a significant increase in β2YFP fluorescence with increasing concentrations of RIC-3 (p < 0.0001, Kruskal-Wallis rank sum test) (Figure 4C).

Therefore, the effect of RIC-3 on α4β2 receptors is vastly different than α7 nAChRs. RIC-3 does not affect subunit assembly between α4 and β2 but upregulates α4 and β2 protein expression.

### RIC-3 interacts with α7 and β2 subunits

We have shown that RIC-3 increases assembly of α7 but not α4β2 nAChRs and that high concentrations of RIC-3 at 5:1 can disrupt α7 and α4β2 assembly. Using FRET measurements between a fluorescently tagged RIC-3 (CFP-RIC-3) and one of the fluorescently tagged nicotinic receptors we examined whether RIC-3 selectively interacts with α7 receptors.

**Figure 5:**
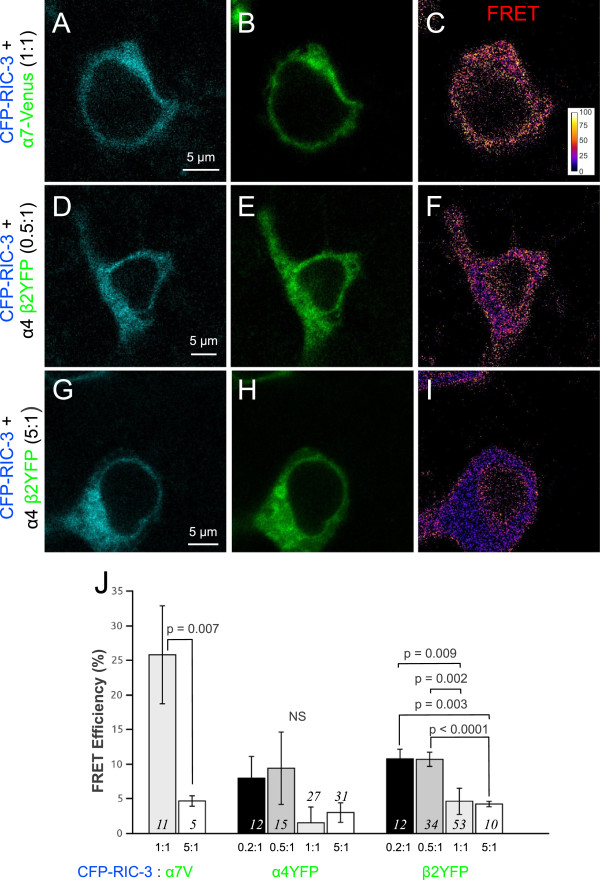
**FRET efficiency measurements show that RIC-3 interacts with α7 and β2 nicotinic subunits.** (**A**-**I**) Pixel based FRET was used to monitor assembly between CFP-RIC-3 and a fluorescently tagged nicotinic receptor subunit (either α7-Venus or β2YFP). (**J**) Summary data showing significantly higher levels of FRET efficiency between CFP-RIC-3 and α7-Venus at 1:1 ratio as compared to 5:1 ratio (p = 0.007, Welch two sample *t*-test). There was significantly greater FRET efficiency between CFP-RIC-3 and β2YFP at 0.2:1 (p = 0.003, Wilcoxon signed rank test post-hoc analysis) and 0.5:1 (p < 0.0001, Wilcoxon signed rank test post-hoc analysis) ratios as compared to 5:1 ratio. Also there was significantly greater FRET efficiency between CFP-RIC-3 and β2YFP at 0.2:1 (p = 0.009, Wilcoxon signed rank test post-hoc analysis) and 0.5:1 (p = 0.002, Wilcoxon signed rank test post-hoc analysis) ratios as compared to 1:1 ratio. No significant (NS) FRET efficiency could be detected between CFP-RIC-3 and α4YFP (p = 0.55, Kruskal-Wallis rank sum test) even though there was a similar trend as β2YFP. Therefore, equimolar RIC-3 interacts with α7 while RIC-3 optimally interacts with β2 at a 0.5:1 molar ratio.

In experiments where a 1:1 molar ratio of CFP-RIC-3 was cotransfected with α7-Venus in HEK293T cells there was a high FRET efficiency (26 ± 7%) (Figure 5). In contrast, when a high molar ratio of CFP-RIC-3 was cotransfected with α7-Venus at 5:1, FRET efficiency significantly declined to 4.7 ± 0.8% (p = 0.007, Welch two sample *t*-test).

We examined for interactions between RIC-3 and β2 by transfecting cells with CFP-RIC-3, β2YFP and nonfluorescent α4 subunit cDNA. In contrast to our results for α7, we detected very little FRET at either 1:1 (4.6 ± 1.9%) or 5:1 (4.2 ± 0.4%) CFP-RIC-3 to β2YFP nicotinic subunit ratios. We then tried two lower concentrations of CFP-RIC-3 to β2YFP nicotinic subunit ratios and found significant increases in FRET signal at 0.5:1 (10.7 ± 1.0%) (p < 0.0001, Wilcoxon rank sum test post-hoc analysis) and 0.2:1 (10.8 ± 1.4%) (p = 0.003, Wilcoxon rank sum test post-hoc analysis) (p = 0.0002, Kruskal-Wallis rank sum test) as compared to 5:1. We also found significant increases in FRET at 0.5:1 (p = 0.002, Wilcoxon rank sum test post-hoc analysis) and 0.2:1 (p = 0.009, Wilcoxon rank sum test post-hoc analysis) (p = 0.0002, Kruskal-Wallis rank sum test) in relation to 1:1. In an analogous set of experiments with cells cotransfected with CFP-RIC-3, α4YFP and nonfluorescent β2 subunit cDNA we found a similar trend as with β2YFP with FRET efficiencies increasing as we lowered the CFP-RIC-3 to α4YFP ratio to 0.5:1 (9.4 ± 5.2%) or below (8.0 ± 3.1%, at 0.2:1), while there was very low FRET efficiency at 1:1 (1.5 ± 2.3%) and 5:1 (3.0 ± 1.4%). However, the changes in FRET efficiencies were not significant (p = 0.55, Kruskal-Wallis rank sum test).

These results show that RIC-3 associates with α7 nicotinic receptor subunits and β2 subunits. Interestingly, RIC-3 interacts with α7 and β2 subunits at different relative cDNA ratios – 1:1 with α7 and 0.5:1 with β2.

### Acute nicotine upregulates FRET between α4 and β2 subunits but RIC-3 prevents nicotine-induced upregulation

Chronic nicotine exposure is known to upregulate high affinity nicotinic receptors whether performed *in vivo* in rodents or *in vitro* in cell lines [1,36,37]. The mechanism for receptor upregulation is unclear but many have been proposed [1-4,9,10]. We previously reported increased FRET efficiency between α4YFP and β2CFP nicotinic subunits and increased protein levels of α4YFP and β2CFP subunits in cultured midbrain neurons following 24 hrs of chronic nicotine exposure [1]. In that study we proposed that enhanced receptor assembly may be one contributing mechanism of nicotinic receptor upregulation. However, an equally likely mechanism of increased FRET with 24 hrs of nicotine could involve a decreased degradation of receptors. Nevertheless, both mechanisms are not mutually exclusive and both can potentially contribute to receptor upregulation. Given that the turnover rate for α4β2 nAChRs is relatively slow (half life = 7–13 hrs, [2,9,10,38]), examining the effects of nicotine at a very acute time point (30 min nicotine exposure) would preclude the effects of degradation of the subunits so that any FRET increase, if any, can be attributed to stimulation of assembly of α4β2 nAChRs. Therefore, we examined whether nicotine exposure truly stimulates receptor assembly by looking at a very acute time point, 30 min of nicotine exposure.

**Figure 6:**
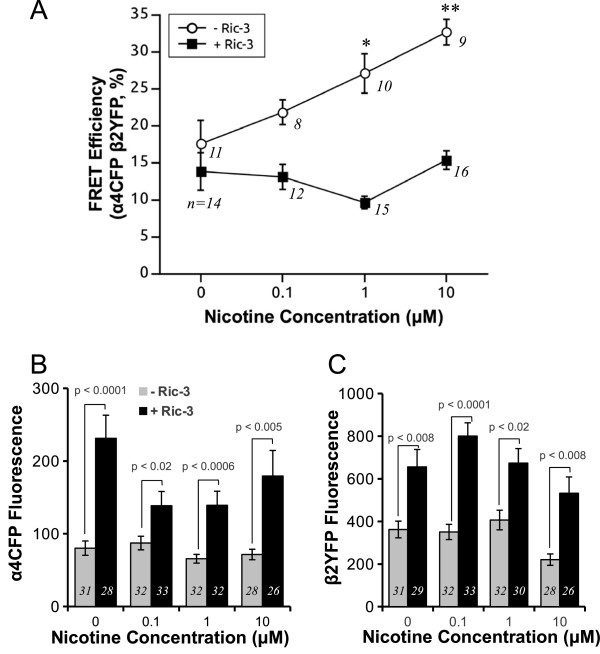
**Effect of acute nicotine treatment on FRET efficiency and protein expression of α4CFP and β2YFP subunits in the presence or absence of RIC-3.** Equimolar amounts of α4CFP and β2YFP cDNA were transfected either with (1:1 ratio of RIC-3 to nAChR subunit) or without RIC-3. Cells were incubated in various nicotine concentrations (0, 0.1, 1 and 10 μM) at 37°C 30 min before imaging. (**A**) In the absence of RIC-3 there was a significant progressive increase in FRET efficiency between α4CFP and β2YFP subunits, signifying receptor assembly, at all concentrations of nicotine (p = 0.002, Kruskal-Wallis rank sum test). With RIC-3 there was no change in FRET efficiency between α4CFP and β2YFP subunits with increasing concentrations of nicotine. Significant difference levels comparing groups of nicotine concentrations relative to no nicotine control: *, p = 0.03, **, p = 0.002 (Wilcoxon rank sum tests). Quantification of mean α4CFP (**B**) and β2YFP (**C**) fluorescence intensities per cell showed no change with increasing nicotine concentrations whether RIC-3 (at 1:1 ratio) was present or absent. However, the mean α4CFP and β2YFP fluorescence intensities with RIC-3 was significantly greater than without RIC-3 at all nicotine concentrations (p < 0.0001, RIC-3 factor, two-way ANOVA; for both α4CFP and β2YFP subunits). For pairwise comparison of α4CFP and β2YFP fluorescence between no RIC-3 and RIC-3 coexpression at each nicotine concentration significance levels are reported as p values (post-hoc pairwise Tukey’s HSD tests).

We first examined changes in FRET efficiencies between α4CFP and β2YFP subunits in the absence of RIC-3 at various doses of nicotine for 30 min exposure. We were surprised to observe a robust increase in FRET efficiency between α4CFP and β2YFP as early as 30 min post nicotine exposure (Figure 6A). This effect was significant (p = 0.002, Kruskal-Wallis rank sum test) and dose-dependent, with significant increases in FRET efficiency at 1 μM (27 ± 3%, p = 0.03, Wilcoxon rank sum test post-hoc analysis) and 10 μM nicotine (33 ± 2%, p = 0.002, Wilcoxon rank sum test post-hoc analysis) as compared to control (18 ± 3%).

In our previous experiments (Figure 4B, C), we discovered that RIC-3 can increase α4CFP and β2YFP protein expression. Therefore, to examine whether RIC-3 could potentially augment the nicotine-mediated FRET increase, we performed the same experiments but additionally coexpressed RIC-3 with α4CFP and β2YFP nAChR subunits. We used the 1:1 ratio of RIC-3 to nAChR subunit because this concentration resulted in a significant and robust upregulation of α4 protein and also was the concentration which maximally increased α7 assembly. Interestingly, there was no change in FRET efficiency as RIC-3 prevented nicotine-mediated upregulation of α4CFP β2YFP receptor assembly at all concentrations of nicotine tested (Figure 6A). However, regardless of the absence or presence of nicotine at various concentrations, RIC-3 was still effective at significantly increasing α4 and β2 protein expression (p < 0.0001, RIC-3 factor, two-way ANOVA; for both α4 and β2) as measured from α4CFP and β2YFP fluorescence (Figure 6B, C). However, 30 min of nicotine at all concentrations was ineffective at altering α4CFP or β2YFP protein levels as measured by fluorescence intensity whether RIC-3 was present or not (Figure 6B, C).

Therefore, coexpression of the heteromeric α4β2 nicotinic receptors with RIC-3 inhibits the effect of nicotine on upregulating FRET between receptor subunits. However, RIC-3 specifically upregulates α4 and β2 subunit protein levels regardless of the presence of nicotine.

### Anti-HA epitope receptor binding confirms that RIC-3 but not 30 min nicotine upregulates α4 and β2 protein

**Figure 7:**
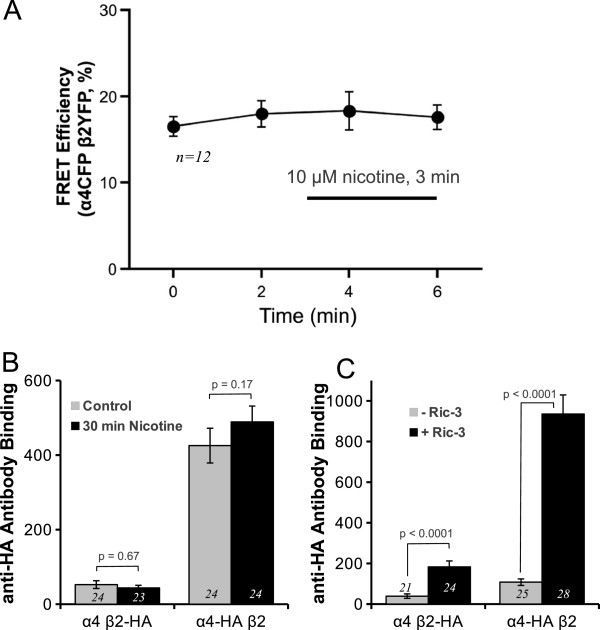
**Anti-HA epitope antibody binding confirms no change in nAChR protein with 30 min nicotine and no change in nAChR conformation within 3 min of nicotine.** (**A**) Equimolar amounts of α4CFP and β2YFP cDNA were transfected in HEK293T cells and time lapse imaging of FRET was performed at 30°C. Cells were imaged for FRET at 1 and 3 min before and 1 and 3 min during 10 μM nicotine incubation. There was no significant change (p = 0.88, one-way repeated measures ANOVA, n = 12) in FRET efficiency between nicotinic receptor subunits with bath applied nicotine during the 3 min nicotine incubation. (**B**) 30 min application of 10 μM nicotine did not alter anti-HA epitope antibody binding of HA epitope tagged α4 (α4-HA) and β2 (β2-HA) subunits expressed in HEK293T cells. (**C**) RIC-3 coexpression with α4-HA and β2-HA subunits resulted in significantly enhanced levels of anti-HA antibody labelling of α4-HA (p < 0.0001, Wilcoxon rank sum test) and β2-HA (p < 0.0001, Wilcoxon rank sum test) subunits as compared to cells not coexpressing RIC-3.

We showed that 30 min of nicotine incubation increased FRET efficiency between α4CFP and β2YFP (Figure 6A), which we interpret as increased subunit assembly. However, one possibility is that the changes in FRET may reflect a conformational change in the receptors due to decreased distance between subunits during channel gating with nicotine and may not have anything to do with increased receptor assembly. To rule out this possibility, we performed a set of FRET experiments to examine whether nicotine can cause a conformational change to the receptor that is sensed by our FRET assay (Figure 7A). This should occur less than a second following nicotine binding to the receptor and result in a conformational change. Therefore, we performed time lapsed FRET measurements between α4CFP β2YFP to monitor FRET changes over a very brief period, less than 3 min of bath applied 10 μM nicotine, which should be long enough to monitor any FRET changes due to conformational changes of the receptors as they bind to nicotine but too brief to monitor any receptor assembly. Accordingly, we measured baseline FRET efficiencies, then bath applied 10 μM nicotine and then monitored FRET at 1 and 3 min during nicotine application. Our results show that at a time scale of 3 min there is no significant FRET change between α4CFP and β2YFP (p = 0.88, one-way repeated measures ANOVA, n = 12) (Figure 7A). These results show that the FRET measurements that we undertook are not complicated by FRET changes due to receptor conformational changes at 3 min nicotine exposure. Furthermore, we do witness a robust FRET increase at 30 min with nicotine incubation (Figure 6A). Together, these results support our hypothesis that nicotine is enhancing receptor assembly (over 30 min of nicotine exposure).

Although in a previous study we have shown that following 24 hrs of nicotine incubation there is upregulation of α4 and β2 subunit protein expression [1], in this study we could not detect changes in protein expression of α4CFP and β2YFP after 30 min of nicotine exposure as measured by fluorescence from the fluorescent protein tags. To verify these results we repeated analogous experiments using a concentration of nicotine (10 μM) that resulted in maximal nicotine-induced upregulation of receptor assembly and examined whether there was an upregulation in α4 and β2 subunit protein expression using anti-HA antibody labelling of the nicotinic receptor subunits. We used either a combination of α4CFP β2YFP or α4YFP β2CFP transfected in cells. β2YFP and α4YFP each contained an HA epitope immediately upstream of the fluorescent protein and were identified in Figure 7 as β2-HA and α4-HA, respectively. Similar to our previous results (Figure 6B, C), 10 μM nicotine for 30 min did not significantly alter α4 (p = 0.17, Wilcoxon rank sum test) nor β2 (p = 0.67, Wilcoxon rank sum test) subunit protein levels (Figure 7B). However, our anti-HA binding assay was able to detect significant increases of α4 (p < 0.0001, Wilcoxon rank sum test) and β2 (p < 0.0001, Wilcoxon rank sum test) subunit proteins with RIC-3 cotransfection (Figure 7C).

These set of data using a second experimental approach are consistent with our previous data and support that 30 min of nicotine exposure does not result in any detectable change in α4 and β2 subunit protein expression. However, with over two days of coexpression with RIC-3 results in an augmentation of α4 and β2 protein levels. Furthermore, the fact that we see nicotine induced increased in FRET over 30 min and not within 3 min supports that our FRET increases reflect receptor assembly and rules out FRET changes due to nicotine induced conformational changes of the receptor as a result of channel gating. However, Govind et al. [38] showed that there are two time courses of nAChR upregulation. Their results showed that the rapid upregulation (time constant = 1.1 hrs) component is due to receptor conformational changes. Thus, we cannot rule out that the FRET changes between α4β2 with 30 min nicotine may be contributed by receptor conformational changes independent of channel gating.

### Acute nicotine does not upregulate assembly of α7 receptors in the presence or absence of RIC-3

**Figure 8:**
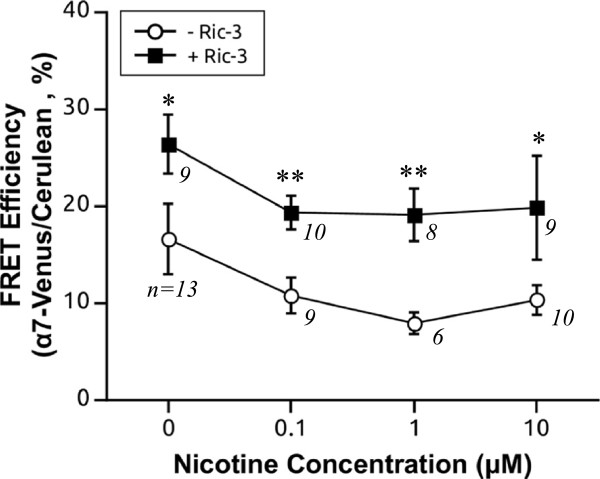
**Effect of acute nicotine treatment on FRET efficiency of α7-Cerulean and α7-Venus subunits in the presence or absence of RIC-3.** Equimolar amounts of α7-Venus and α7-Cerulean were transfected either without RIC-3 or with RIC-3 at a 1:1 ratio to the nAChR subunit. Cells were incubated at various concentrations of nicotine (0, 0.1, 1 and 10 μM) at 37°C for 30 min prior to imaging. Whether RIC-3 was present or absent, there was no change in FRET efficiency between α7-Venus and α7-Cerulean subunits over increasing concentrations of nicotine (30 min) (p = 0.3, and p = 0.12, Kruskal-Wallis rank sum tests, respectively). However, the FRET efficiency between α7-Venus and α7-Cerulean was signficantly greater in the presence than the absence of RIC-3 at each of the nicotine concentrations (*, p < 0.05, **, p < 0.01, Wilcoxon rank sum test or *t*-test).

It has been proposed that nicotine acts as a pharmacological chaperone to enhance assembly of α4β2 nicotinic receptors [9]. To determine if agonist binding can also enhance assembly of α7 subunits, the effect of acute (30 min) nicotine treatment on receptor assembly was tested, first in the absence of the chaperone, RIC-3. We found that varying the concentrations of nicotine from 0.1 to 10 μM did not change the FRET efficiency between α7-Cerulean and α7-Venus in the absence of RIC-3 (p = 0.12, Kruskal-Wallis rank sum test) indicating that acute nicotine was unable to upregulate α7 receptor assembly (Figure 8).

We surmised that the lack of effect of nicotine on α7 may have been due to the fact that there were no surface receptors to begin with in the absence of RIC-3 and that there would need to be a basal level of functional surface receptors for acute nicotine to have its effect. Hence, we repeated the same experiments but this time we coexpressed RIC-3 in a 1:1 ratio with α7-Cerulean and α7-Venus in order to examine the effect of acute nicotine in the presence of assembled α7 receptors. Similarly, following 30 min nicotine incubation we witnessed no change in FRET efficiency between α7-Cerulean and α7-Venus subunits over all concentrations of nicotine tested vs control (Figure 8) (p = 0.4, one-way ANOVA). However, at all nicotine concentrations α7 receptors had significantly higher FRET efficiencies with RIC-3 as compared to without RIC-3 (p = 0.001, RIC-3 factor, two-way ANOVA) (Figure 8), suggesting that RIC-3 can still enhance receptor assembly independent of the presence of nicotine. Notably, nicotine had no effect on α7 subunit expression as there was no significant change in mean α7-Cerulean and α7-Venus fluorescence per cell at all nicotine doses tested, whether RIC-3 was present or not (data not shown).

Therefore, acute nicotine treatment does not upregulate assembly of α7 receptors, and RIC-3 co-transfection is insufficient to promote the sensitivity of the receptor to upregulation by the agonist.

## Discussion

In the present study we examined the role of RIC-3 chaperone protein on the assembly of α7 and α4β2 nAChRs and the effect of RIC-3 in modulating nicotine mediated upregulation of α4β2 and α7 receptor assembly. Using FRET analysis between fluorescently tagged α7 subunits we found that low RIC-3 concentrations increased assembly of α7 receptors in a dose-dependent manner, but had no effect on receptor assembly at high RIC-3 concentrations (5:1 ratio of RIC-3 to α7). This was corroborated with Fl-Bgt labeling, which additionally showed that RIC-3 stimulated forward trafficking of the receptor to the cell surface at all concentrations. In contrast, the chaperone did not affect assembly of the heteromeric α4β2 receptors, suggesting that the effect of RIC-3 on assembly is specific to the homomeric α7 receptor. However, RIC-3 specifically upregulated α4 and β2 subunit protein expression in a dose-dependent manner, but had no effect on α7 subunit protein expression. We examined the effects of acute nicotine exposure and found that 30 min was sufficient to stimulate upregulation of FRET between α4 and β2 receptor subunits in a nicotine dose-dependent manner (0.1 – 10 μM) in the absence of RIC-3. Interestingly the presence of RIC-3 prevented nicotine-mediated upregulation of FRET between α4 and β2 at all nicotine concentrations. Acute nicotine exposure (30 min) was unable to upregulate α7 nicotinic receptors whether or not RIC-3 was present. At this short time frame (30 min) of nicotine exposure, there was expectantly no detectable change in α4, β2 and α7 protein expression.

### RIC-3 increases assembly and surface expression of α7 receptors

FRET is a spectroscopic technique that can be used to measure receptor assembly or receptor subunit stoichiometry [1,27,28,39]. The marked enhancement in FRET efficiencies between the fluorescent α7 subunits with increasing amounts of RIC-3 plasmid (Figure 1) supports previous studies that RIC-3 acts as a chaperone to promote the maturation and assembly of α7 receptors [17,22,23,35]. Assembly levels peaked when the RIC-3 chaperone and the receptor were transfected at equimolar ratios suggesting that the stoichiometry for efficient receptor assembly is one RIC-3 molecule for every α7 subunit [20].

Interestingly, FRET between α7 subunits returned to control values at a 5:1 transfection ratio (Figure 1), suggesting that at high expression levels RIC-3 loses its ability to mediate receptor assembly likely due to RIC-3 self aggregation [22]. At sufficiently high chaperone concentrations in the ER, RIC-3 monomers may self-assemble, likely via their coiled-coil domains [40], leading to the formation of large aggregates. Such conditions would sterically hinder the ability of RIC-3 to effectively bring α7 subunits into close proximity, preventing assembly and retaining the subunits in the ER. This is supported by our FRET experiments between CFP-RIC-3 and α7-Venus, which showed a significantly high FRET efficiency when they were coexpressed at 1:1 ratio and a negligibly low FRET efficiency when expressed at 5:1 (Figure 5). We think that at high RIC-3 concentration (5:1) RIC-3 does not lose its ability to interact with α7. A likely possibility is that the FRET signal is diluted at 5:1. At 5:1 CFP-RIC-3 to α7-Venus, RIC-3 is in excess. For every RIC-3 bound to α7-Venus there are four molecules of RIC-3 that are unbound. Because FRET signal relies on the interaction of a donor fluorescent molecule to its acceptor, we would predict that the 5:1 ratio should have a FRET efficiency that is 20% of that at 1:1 ratio because of excess unbound donors. This is what we observe as at 1:1 the FRET efficiency is 26 ± 7% and at 5:1 the FRET efficiency is 4.7 ± 0.8%, which is 18% of 1:1 and is within the standard error range.

Results from α-Bgt labelling experiments were used to support the FRET measurements of receptor assembly. The Fl-Bgt signal was almost undetectable in the absence of RIC-3, but the total number of Fl-Bgt binding sites increased significantly in the presence of the chaperone (Figures 2, 3). In agreement to the initial FRET measurements (Figure 1J), this result further demonstrates that RIC-3 can increase assembly of α7 subunits. It was important, however, to confirm that the increase in the total number of α-Bgt binding sites was due to assembly of already expressed subunits rather than an increase in protein levels. In agreement with previous studies [22,41] the protein levels of α7 did not change significantly upon cotransfection with the chaperone (Figure 3). Thus, the effect of RIC-3 on subunit assembly is entirely independent on expression levels of individual subunits. It was found that the number of α-Bgt binding sites increased more extensively on the surface than within the cell upon addition of RIC-3 (Figure 3). Alexander and colleagues [22] have observed a similar trend, with a 5-fold increase in whole-cell levels, and a 40-fold increase in surface levels of receptor upon cotransfection of the chaperone. Thus, RIC-3 has an additional role of transporting the receptor to the surface. In fact, since proper assembly is a pre-requisite for exit of the receptor from the ER [42] the sole ability of RIC-3 to enhance receptor assembly could promote its release from the ER and procession along the secretory pathway.

Interestingly, the highest level of RIC-3 transfection (5:1 ratio to α7) dropped surface expression levels relative to the peak values, although these levels were still significantly higher than control. Again, this data suggests that RIC-3 can partly lose its function when expressed at high levels, possibly by forming self-aggregates in the ER that hinder α7 export to the surface. In contrast, whole cell levels of receptors peaked at this transfection ratio, which suggests that although the aggregates may hinder receptor trafficking, they have no deleterious effects on its assembly. Our Fl-Bgt data are consistent with the results of the study of Alexander and colleagues [22]. However, the α-Bgt labelling data differ somewhat to our FRET results, which indicate a loss of chaperone-mediated assembly at the highest level of RIC-3 (Figure 1J). Alexander and colleagues [22] observed the formation of aggregates containing both RIC-3 and α7 in structures they described as autophagosomes in high expressing RIC-3 cells. At high RIC-3 concentrations α7 did not traffic to the cell surface but was retained in the ER and accumulated in aggregates. Another study showed that autophagosomes are responsible for degradation of AMPA receptors [43]. Perhaps there could be degradation of the excess assembled nicotinic receptors in the autophagosomes but the overall number of receptors are still elevated. This would be consistent with an increase in total α-Bgt binding sites that we observed in Figure 3A. Since FRET measures the proportion, not the total number, of assembled subunits and high RIC-3 is possibly stimulating the degradation of excess assembled nicotinic receptors, a change in proportion would decrease the overall FRET signal closer to control values, which is what we observe at 5:1 RIC-3 to α7 (Figure 1J), regardless of the net increase in total assembled receptors, which is what we observed with α-Bgt labelling (Figure 3A). Interestingly, we observed this similar trend with α4β2, where at 5:1 RIC-3 to α4β2 there was a decrease in FRET efficiency between α4 and β2 subunits (Figure 4A). At 5:1 RIC-3, the reason that there is no change in α7 subunit protein levels is that there may be an increased production of unassembled α7 protein subunits that balances the degradation of α7 receptors, which will further decrease FRET efficiency.

### Effects of RIC-3 on α4β2 receptors

FRET between α4 and β2 constructs does not change significantly when RIC-3 is coexpressed with the heteromeric subunits (Figure 4A) at all concentrations of RIC-3 except for the high 5:1 concentration, which attenuated FRET signal. This suggests that the ability of RIC-3 to enhance receptor assembly is specific to α7. Interestingly, using FRET measurements between CFP-RIC-3 and fluorescently tagged nAChR subunits, we observed that RIC-3 also interacts with β2YFP but at a lower cDNA ratio than with α7-Venus. Through FRET we observed that RIC-3 interacts with β2YFP at a 0.5:1 ratio while with α7-Venus RIC-3 interacts at a 1:1 ratio (Figure 5). We observed a similar trend of FRET efficiencies between CFP-RIC-3 and α4YFP over various concentrations of CFP-RIC-3 but the results did not reach significance. These results are supported by a previous study [17], which showed that both α4 and β2 nAChR subunits interact with RIC-3 using immunoprecipitation experiments. Although a lower cDNA ratio of RIC-3 was required to interact with β2 as compared to α7, future experiments must be performed to determine whether the stoichiometry of interaction at the protein level is actually lower for β2.

Although we did not find that RIC-3 stimulated any changes in assembly between α4 and β2 subunits, we did find that RIC-3 specifically increased α4 and β2 subunit protein expression (Figures 4 and 6), which would likely result in increased nicotinic responses given that the proportion of assembled receptors would remain unaltered but the total number of assembled receptors would increase. This is supported by the same previous study which showed that RIC-3 steadily increases functional expression of the α4β2 receptors in transfected mammalian cells [17].

Furthermore, the ability of the chaperone RIC-3 to increase α4 and β2 subunit protein expression makes sense, given that the association of other chaperone proteins such as 14-3-3 and phosphorylation of α4 results in increased expression of α4β2 receptors as determined by cytisine binding and Western blots [44]. In comparison to other subunits, α4 has the largest M3-M4 cytoplasmic loop (~260 aa) with many post-translational modification sites that can potentially regulate protein expression and receptor turnover [1,44,45]. It is conceivable that when RIC-3 binds to α4β2 receptors RIC-3 may act like 14-3-3 to upregulate receptor protein expression levels [45] possibly by inhibiting signals targeting for degradation. In fact, β2 subunits have a strong ER retention motif and by binding to RIC-3, this may stimulate forward trafficking of α4β2 receptors and thus, protect them from ER associated degradation [22]. However, further studies are required to reveal the exact mechanism by which RIC-3 modulates protein levels of these subunits. We expect that the total number of assembled receptors would increase because RIC-3 maintains the same proportion of assembled α4β2 receptors as measured by no change in FRET while the protein levels of both subunits increase.

The fact that RIC-3 does not influence assembly of α4β2 receptors is expected. In fact, maturation of the heteromer may rely on other chaperones, such as Calnexin, Bi P, UNC-50, and 14-3-3 [45-47]. Furthermore, unlike α7, heteromeric α4β2 receptors can express at high levels in a variety of systems in the absence of RIC-3 [17] suggesting that the chaperone is not essential for the assembly of α4β2. The ability of RIC-3 to have differing effects on specific receptor subtypes may have physiological implications. α7 homomers are only observed in regions of the brain that also express RIC-3 [48]. By regulating the tissue-dependent expression of RIC-3, the nervous system can directly and specifically control the regional distribution of α7 expression in the brain, without altering the expression levels of other receptors.

### RIC-3 modulates nicotine-induced receptor upregulation

The majority of studies on nicotine-mediated nAChR upregulation have focused on the chronic effects of agonists or antagonists, mainly on time frames of 24 hrs to a few days in cell and neuronal culture [1-4,9-11], 10 to 30 days for chronic nicotine exposure in rodents [6,7,36,49] and many years in smokers’ brains [8,50]. Multiple parallel mechanisms of nicotine-induced nicotinic receptor upregulation are likely to exist [38]. Harkness and Millar [51] showed that α4β2 receptors increase in total and surface epibatidine binding after 24 hrs of nicotine exposure but there was no change in overall protein levels of α4 or β2. Post-trancriptional or post-translational mechanisms must be involved in receptor upregulation since there is no change in mRNA transcript for both α4 or β2 [7]. A plausible explanation of increased epibatidine binding with no subunit protein change could be increased receptor assembly of an already existing pool of unassembled receptor subunits. We previously found that 24 hrs of nicotine exposure increased FRET between α4 and β2 receptors indicating that there were more assembled receptors [1]. We proposed that 24 hrs of nicotine stimulates assembly of the receptors. However, an equally likely scenario is that 24 hrs of nicotine protects the receptors from degradation [52]. The half-life of α4β2 receptors is reported to be 7–13 hrs [2,9,10]. However, our results show increased FRET efficiency between α4 and β2 subunits after 30 min of incubation with nicotine (Figure 6A). This finding supports that nicotine is stimulating assembly of receptors since a decreased degradation rate cannot account for the alteration in receptor numbers at this short time frame of 30 min. The effect of nicotine decreasing the degradation rate of receptor protein would require hrs to be detectable and is also likely to contribute to receptor upregulation at the longer time scale of hrs [38].

However, one potential caveat in our FRET experiments between α4CFP and β2YFP is that nicotine would cause conformational changes due to gating of the α4β2 channels, which would result in changes in FRET. When we applied nicotine in the dish we witnessed no alterations in FRET at 1 and 3 min during nicotine application and therefore ruling out this possibility (Figure 7A). Since the fluorescent proteins are in the cytoplasmic loops, a region not known for gating, FRET changes were not expected. This set of control experiments support our hypothesis that 30 min of nicotine is stimulating assembly. However, Govind and colleagues [38] showed that nAChR upregulation has two different mechanisms occurring at different rates. Their results support that the fast transient (τ = 1.1 hrs) nAChR upregulation corresponds to nAChR conformational changes. Our FRET experiments cannot rule out the possibility that our nicotine-induced increase in FRET between α4β2 may be contributed by receptor conformational changes.

Our results also showed that 30 min of nicotine stimulated increased FRET between α4 and β2 with no change in both α4 or β2 subunit protein expression (Figures 6B, C, 7B). However, at longer time scales we found that 24 hrs increased α4 and β2 protein expression in cultured midbrain neurons and 10 days of chronic nicotine exposure in mice increases α4 subunit protein expression in the CNS [6,53]. This is likely due to the fact that mRNA transcription, followed by protein translation would require several hours to manifest, and thus cannot occur within the time frame of the present experiments.

Surprisingly, when we coexpressed RIC-3 with α4β2 receptors, 30 min nicotine was unable to upregulate receptor assembly (Figure 6A), suggesting that RIC-3 prevented nicotine-induced receptor upregulation of assembly. This data opens the possibility that RIC-3 can modulate α4β2 receptor upregulation in specific neurons in the CNS by the selective co-expression of RIC-3. Notably, the phenomenon of nicotine-induced receptor upregulation occurs in many but not all regions of the brain [5-7]. We have shown previously that chronic nicotine can cause selective upregulation in specific subtypes of neurons in particular brain regions [6]. Specifically in the VTA and SN α4 receptors are upregulated in GABAergic neurons but not dopaminergic neurons. We propose a novel mechanism whereby nicotine-mediated receptor upregulation can be prevented by the effects of the RIC-3 chaperone protein. However, there are likely other possibilities including the presence of other nicotinic receptor subunits, such as α5, with α4β2 receptors can inhibit receptor upregulation *in vivo*[54]. Furthermore, in mouse brains following chronic nicotine administration nicotine-induced nAChR upregulation occurs in many brain regions except for specific brain regions where α4 is highly expressed such as the medial habenula, thalamus and dopaminergic neurons where there is no nicotine-induced upregulation of receptors [6,7]. These reported data may be analogous to the effect of RIC-3 preventing nicotine-induced upregulation. A possibility is that the receptor number in both scenarios are already at a high and saturated level so nicotine cannot further increase protein levels (Figures 6B, C, 7C). This hypothesis is supported by a previous study by Wang and colleagues [55] who showed that cells expressing α3β2 had low levels of receptors but had robust nicotine-induced upregulation. In contrast, cells expressing α3β4 receptors had approximately four times greater baseline receptor expression than α3β2 but nicotine exposure was unable to upregulate α3β4 receptors.

The fact that nicotine, at all concentrations tested, did not induce upregulation of α7 receptor assembly is not surprising. In general, α7 receptors are more resistant to nicotine-induced upregulation. In cell lines, α7 receptors require at least 1000 fold higher concentration of nicotine (10 μM vs 10 nM) than α4β2 and α7 receptors show a minimal amount of upregulation as compared to α4β2, which may show several fold upregulation in cell lines [4,11,12]. In studies on human smokers’ brains, there is even conflicting data as to whether α7 receptors are increased at all unlike α4β2, which are consistently augmented in smokers’ brains [8,50,56]. Several studies have noted increased expression of α7 receptor levels following treatment with agonists, but these trends are only noted in incubation periods longer than 24 hours [11-13]. Since nicotine may primarily exert its effects on the cell surface, it is conceivable that by increasing the surface expression of mature receptors, RIC-3 may in turn enhance the sensitivity of the receptor to the effects of the agonist. Interestingly, although RIC-3 increased FRET efficiencies between α7 subunits, nicotine treatment at all doses yielded no significant changes in α7 assembly in the presence of the RIC-3 chaperone (Figure 8). These sets of results suggest that one of the mechanisms of nicotine-induced receptor upregulation is stimulation of receptor assembly for α4β2 but not for α7 receptors.

## Conclusions

These results support previous data on the role of RIC-3 in enhancing assembly and surface trafficking of α7 receptors. Additionally, this study demonstrates for the first time that RIC-3 differentially affects assembly and protein expression of α4β2 and α7 receptors. While RIC-3 directly interacts with α7 subunits in a 1:1 cDNA ratio to enhance receptor assembly without altering protein expression, RIC-3 interacts with β2 at a lower cDNA ratio (0.5:1) but does not influence assembly between α4 and β2 subunits. Interestingly, RIC-3 causes a selective increase in expression of α4 and β2 subunits but prevented nicotine-induced increased FRET between α4 and β2 subunits. Since experiments were conducted on HEK293T cells, further investigation would be required to examine whether similar regulation of nicotinic receptor assembly and expression with RIC-3 and nicotine would be recapitulated in subtype specific neurons in the brain. Our results suggest that RIC-3 chaperones could potentially impact addictive behaviors by inhibiting nicotine-mediated α4β2 receptor upregulation of receptor assembly.

## Abbreviations

ACh: Acetylcholine; Fl-Bgt: Alexa Fluor 637 α-bungarotoxin; FRET: Förster resonance energy transfer; nAChRs: Nicotinic acetylcholine receptors; RIC-3: **R**esistant to **i**nhibitors of acetyl**c**holinesterase

## Competing interests

The authors declare that they have no competing interest.

## Authors’ contributions

AD designed, conducted and analyzed some of the experiments and wrote portions of the manuscript. PK conducted and analyzed some of the experiments and performed all the revised experiments of the paper. MT and GM conducted and analyzed some of the experiments. GE produced α7-Venus and α7-Cerulean. RN designed and directed the experiments, conducted and analyzed some experiments, designed α7-Venus and α7-Cerulean and wrote the manuscript. All authors read and approved the final manuscript.
